# Stromal tumor of the lesser omentum : a case report

**DOI:** 10.11604/pamj.2014.17.236.3133

**Published:** 2014-03-28

**Authors:** Hossam Behammane, Younes Aggouri, Morad Oussaid, Said Ait Laalim, Imane Toughrai, Karim Ibn majdoub, Khalid Mazaz

**Affiliations:** 1 Département de Chirurgie, Faculté de médecine et de pharmacie de Fès, Université Sidi Mohammed Ben Abdellah, CHU Hassan II, Fès, Maroc

**Keywords:** Lesser omentum, extragastrointestinal stromal tumor, CD117, surgical resection

## Abstract

Gastrointestinal stromal tumors (GISTs) represent the majority of primary non-epithelial neoplasms of the digestive tract, most frequently expressing the KIT protein detected by immunohistochemical staining for the CD117 antigen. Extragastrointestinal stromal tumors (EGISTs), neoplasms with immunohistological features overlapping those of GISTs, are found in the abdomen outside of the gastrointestinal tract with no connection to the gastric or intestinal wall. We report the clinical, macroscopic and immunohistological features of an EGIST arising in the lesser omentum of a 58-year-old woman. This is a very rare location of intra abdominal stromal tumors.

## Introduction

Extra gastrointestinal stromal tumours are the rare intra abdominal tumours which occur in the omentum, the mesentry and other intra abdominal sites. By definition, they display no connection, however tenuous, to the wall or serosal surface of viscera. The EGISTs in the lesser omentum can grow slowly in the abdomen for a long time without clinical appearance. In most cases a preoperative diagnosis is not possible, and the patient undergoes a surgical operation for the generic diagnosis of ″abdominal mass″.

## Patient and observation

A 54-years old woman, operated a 6 months ago for uterine leiomyosarcoma for which he was made a colpohysterectomy. In a balance control was fortuitously discovered an abdominal mass for which she was admitted to our department for treatment. Clinical examination found a patient in good general with the abdominal examination a scar in umbilical median laparotomy, the rest of the physical examination is unremarkable. A biological assessment was conducted, including tumor markers (CEA, CA19-9), were unremarkable. Thoraco-abdominopelvic scanner found a mass of tissue measuring 32/30mm lesser omentum mass suggestive of lymph node or a gastric stromal tumor.

**Figure 1:**
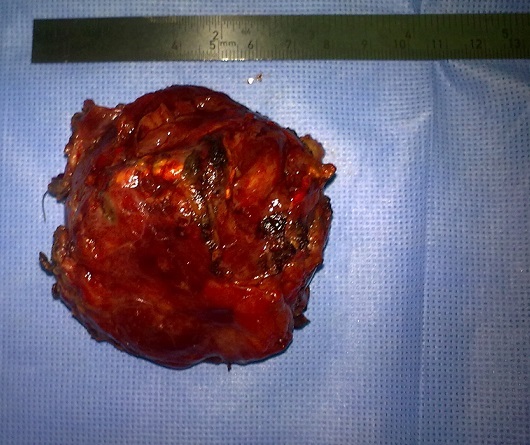
Surgical specimen: tumor lesser omentum

**Figure 2:**
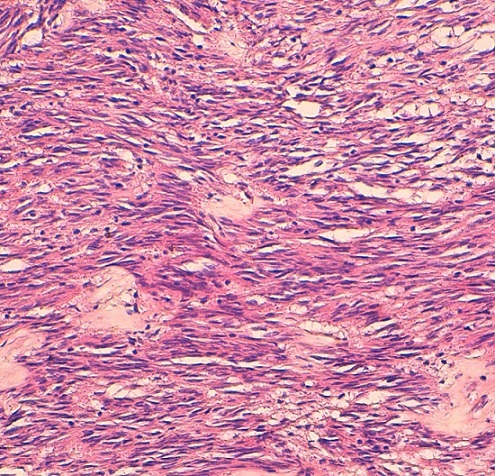
Fusocellulaire dense proliferation arranged chaotically entangled beams(HE × 200)

**Figure 3:**
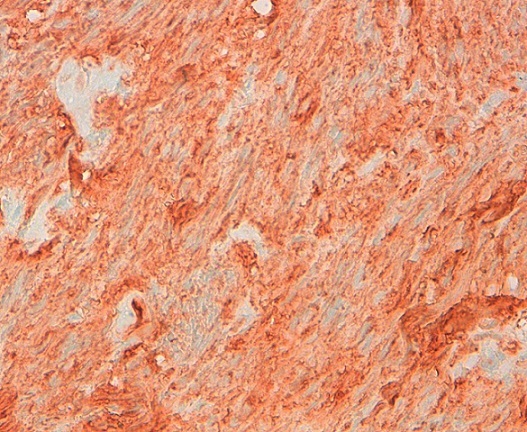
Positive immunomarquge cKit (× 400)

The patient was operated laparoscopically, exploration has shown a tumor depends on the lesser omentum was resected (Figure 1). The postoperative course was uneventful; the patient was put in the outgoing second day of postoperative. Pathological examination returned for a stromal tumor (Figure 2, Figure 3).

## Discussion

The EGIST stromal tumors are developed from the abdominal soft tissue and are considered primitive when they have no connection with the digestive tract [1]. They are much rarer than gastrointestinal stromal tumors (GIST) (less than 10%), much less described in the omentum [2]. In contrast to GIST, EGIST of histogenesis is still poorly understood. The expression of ckit by EGIST suggests the presence of interstitial cells of Cajal outside the gastrointestinal tract or rather the ability of mesenchymal cells to express the same phenotype of aberrantly [1]. Miettinen et al. believe that omental and mesenteric EGIST respective derivatives of the stomach and intestine can become detached during their development [3]. The clinical symptom is often delayed [3]. The contribution of imaging (CT, MRI) is undeniable in the preoperative diagnosis of EGIST [4], it allows, in addition to the visualization of the tumor to guide the biopsy fine needle for diagnostic. According to Ortiz-Rey et al. [5], this action is relatively simple and beneficial in the current indication EGIST.

However, the exact starting point of the process is not always easily determined by imaging, our patient was operated for a tumor of presumed gastric origin, the final pathological examination of surgical specimens not found any connection to the tumor stomach is strictly normal. The morphological appearance immunophenotypical and character are those of GIST. The literature data are conflicting regarding the prognosis of EGIST. SBR grading system used for GIST, combining tumor size and mitotic index [6] cannot be extrapolated for EGIST, the latter being most often large at diagnosis. The seat of the tumor, it seems, an independent prognostic factor [7], the omental EGIST are deemed more aggressive than mesenteric. Yamamoto et al. [8] define three prognostic grades on the basis of mitotic index and Ki67 proliferation index tumor, a mitotic index greater than or equal to 5 / 50 CFG and / or a proliferation index greater than or equal to 10% can to classify the tumor EGIST high risk of malignancy. In contrast, a tumor with a mitotic index of less than 5 / 50 CFG and / or a proliferation index less than 10% is considered low risk of malignancy. The molecular profile of the tumor will be validated in the future as a prognostic factor [9].

The complete surgical resection of the tumor with clear margins is the first-line treatment of metastatic non EGIST [8]. The EGIST overexpress CD117 and present profiles of gene mutation and PDGFRA CKIT similar to GIST. A specific study of Glivec treatment options in this group of EGIST is not feasible given the rarity of this disease. Of EGIST were included in the randomized Phase III evaluated with imatinib in patients with GIST. Treatment with imatinib is entirely lawful and appropriate at this time when locally advanced or metastatic [10].

## Conclusion

The EGIST are rare mesenchymal tumors with morphological and phenotypic similarities with GIST. Their evolutionary potential and the therapeutic possibilities, however, are less well codified by lack of study material.
